# Optimizing laboratory cultivation of wood-inhabiting fungi with emphasis on applied conservation

**DOI:** 10.1007/s00253-025-13603-1

**Published:** 2025-09-30

**Authors:** Joette Crosier, Lorin von Longo-Liebenstein, Mattias Edman, Sylwia Adamczyk, Leena Hamberg

**Affiliations:** 1 Forest Health and Biodiversity Unit, Natural Resources Institute Finland (Luke), Latokartanonkaari 9, 00790 Helsinki, Finland; 2 Soil Ecosystems Unit, Natural Resources Institute Finland (Luke), Latokartanonkaari 9, 00790 Helsinki, Finland; 3 Department of Natural Science, Design and Sustainable Development, Mid Sweden University, 852 30Sundsvall, Mittuniversitetet, Holmgatan, Sweden; 4 Kettula, Finland

**Keywords:** Culture collections, Grain spawn, Sawdust spawn, Supplementation, Temperature, Substrate specificity

## Abstract

**Abstract:**

While fungi have been grown for centuries as food, cultivation knowledge rarely extends to rare fungi, usually confined to those with high biotechnology or food value. A more robust knowledge base on cultivating rare, challenging fungi may be critical for conservation, as many species face extinction. They should be cultivated for gene banks, reintroduction, and other conservation work. This study adapted mushroom growing techniques for seven threatened wood-inhabiting fungi, all red-listed in Finland and Sweden: *Antrodia crassa*, *Antrodia infirma*,* Amylocystis lapponica*,* Skeletocutis stellae*,* Perenniporia tenuis*,* Radulodon erikssonii*, and *Haploporus odorus*. We grew mycelium of these species (five strains each, two for *P. tenuis*) under various laboratory conditions. We tested wood dust supplementation (in agar), grain spawn substrate composition, gas exchange rates, two wood types (natural host and birch) on sawdust and dowel spawn, and temperature range (6.0–36.5 °C). We measured growth rate in all conditions and ergosterol (mycelial biomass indicator) in wood type and wood dust agar tests. We found wood dust–supplemented agar had an overall positive effect. Temperature effects varied by species, with some preferring relatively warm or cool temperatures, and some having a narrower growth range. Most species grew better on grain when vermiculite was added; gas exchange had no effect. Wood type had a variable effect, but birch was suitable in all cases, sometimes better than the natural host wood. Overall, our treatments had positive to neutral effects on mycelial growth of our fungal species.

**Key Points:**

• *Optimized laboratory cultivation methods can benefit fungal conservation and other applied mycology efforts.*

• *Certain supplements for increasing media complexity or retaining substrate moisture lead to improved growth of challenging fungi.*

• *Optimal conditions vary by species and strain, but general guidelines may apply more broadly, and natural habitat conditions can offer a starting point.*

**Supplementary Information:**

The online version contains supplementary material available at 10.1007/s00253-025-13603-1.

## Introduction

Mycological cultivation methods are well developed and have a long history of optimization in the mushroom growing industry (Stamets 2000; Balan et al. 2022). However, these methods have primarily focused on very limited species, including mostly those which have historical edible and commercial value. These cultivated fungi are then often further narrowed down by ease of cultivation, as there is economic incentive for commercial organizations to grow mushrooms which will have low fall-out and high yield with a relatively relaxed margin of error. This limited focus is evident, as five genera—*Lentinula*, *Pleurotus*, *Auricularia*, *Agaricus*, and *Flammulina*—make up 85% of global edible mushroom cultivation (Royse et al. 2017). Some standard mycology lab methods do not work for the rare and more challenging fungi, as species and even individual strains have unique growth requirements. As many fungi face extinction threats (Antonelli et al. 2023), research in fungal conservation is growing (Singh et al. 2018; May et al. 2019). Approaches like reintroduction (Piętka 2005; Piętka and Grzywacz 2006; Abrego et al. 2016; Crosier et al. 2025) and ex situ conservation through living culture collections (Singh 2017) make apparent the need to adapt cultivation methods for a broader range of species, ensuring the ability to preserve them for future uses, including reintroduction.

It is well-established in mushroom cultivation that early-stage conditions affect later success. A healthy starting culture with strongly growing mycelium leads to better fruiting results than one with suboptimal growth (Stamets and Chilton 1983). Cultivation requires expert knowledge to identify healthy cultures, but such expertise is often lacking for rare fungi. This study investigates various growing conditions from Petri dish to final spawn stages, focusing on growth speed and ergosterol content as indicators of mycelium vigor. While no single factor guarantees success, comparing variables will help us determine effective conditions for the seven species studied (detailed in the “Discussion” section) and offer insights for working with other rarely grown and potentially challenging species. The primary objective is to optimize cultivation at each stage for better applied outcomes.

One factor that may influence mycelial growth is the choice of carbon source. Research suggests that switching to a more complex carbon source, such as wood, can slow strain senescence associated with prolonged cultivation on simpler substrates (Stamets and Chilton 1983) and may enhance the production of enzymes important for wood colonization (Mäkelä et al. 2013). Building on this, our study investigates whether sawdust supplementation on agar Petri plates can positively impact the mycelial growth of our target species.

Another key factor in fungi cultivation is temperature, which impacts success across a wide range of species and applications. As with other conditions, the degree of impact varies, with some species and strains requiring a narrower range. Some species seem to have a stronger natural temperature dependent preference, and a warmer or cooler microclimate can have a strong impact on optimal growth (Bains 2025) and function (Edman and Carlsson 2021). However, the ideal laboratory growing temperature of any isolate may not be easy to predict, as with some fungi, it is not directly linked to the source climate (Müller et al. 2019) and within a species, two strains can have optimal temperatures differing by 10 °C (Zervakis et al. 2001). Therefore, a temperature range should be investigated for newly isolated strains to determine optimal growing conditions.

Spawn run, the time for mycelium to fully colonize a substrate, is crucial for successful mushroom cultivation. Slow growth, common with rare fungi in suboptimal conditions, increases the risk of substrate drying out and contamination, requiring intervention (Stamets 2000). Research has attempted to optimize spawn run by adjusting moisture and air exchange (Shen et al. 2008), as both factors influence growth and contamination. Some species like those found on dry, barkless “kelo”—decorticated pine (*Pinus sylvestris*) which has died and decayed very slowly—may benefit less from moisture-retentive substrates, whereas species often found in moister may thrive with higher moisture in the lab. Additionally, increased gas exchange during incubation has been shown to reduce spawn run time, positively affect fruiting, and reduce contamination susceptibility in some edible mushrooms (Donoghue and Denison 1995; Maddau et al. 2002). However, this effect may vary, as fungi in low-oxygen environments, such as heartwood or waterlogged wood, may require less gas exchange (Scheffer 1986). This study will explore the impact of both moisture and air exchange on the growth of our seven target species, considering their specific habitat needs.

Finally, beyond growth optimization, conservation projects are often limited by funding and can benefit from improved cost-effectiveness. Studies typically use inoculum made from the fungi’s host wood, with myceliated wooden dowels being the most common method for wood inoculations (Crockatt 2008); Abrego et al. 2016; Edman et al. 2024; Crosier et al. 2025). While dowels made of the natural wood host are commonly used, this approach may not be scalable for large reintroduction projects. Dowels of specific wood types can be costly or hard to source and may not match standard inoculation equipment. Sawdust spawn, generally considered the more efficient inoculation method when done a larger volumes, can be even more difficult to have custom produced using a specific tree species. Therefore, one of our objectives is to see if viable inoculum of some harder to grow fungi can be obtained with a cheap, widely available wood substrate like birch—with as much success as “preferred,” natural wood types.

### Hypotheses

- H1: Supplementing agar with wood dust will lead to faster growth and a higher quantity of viable mycelium in all our species. There may be a stronger impact on the species which are more challenging to cultivate, slow growing, and prone to stalling.
- H2: Species will have highly variable optimal grow temperatures, but will follow trends where microclimate preferences are known (Martikainen et al. 2000; Kunttu 2007; Runnel et al. 2020). 
- H3: Vermiculite addition to grain as a moisture enhancer will generally improve fungal growth rate, and more so in species preferring moist microclimates (Niemelä et al. 1995; Kotiranta and Niemelä 1996; Martikainen et al. 2000), but both that and gas exchange rate will have a variable and interactive effect on the spawn run time of the fungi.
- H4: In both sawdust and dowels, birch wood will be a comparably good material for getting viable mycelium to use for inoculum as the natural host wood.

## Methods

### Biological material

In this study, we used seven fungal species that are red-listed in Finland and Sweden (Table 1). Species were chosen, in this case, based on their candidacy for conservation reintroductions, as they have well-known history of population dynamics and have already disappeared from many areas. The ability to cultivate them underpins the possibility to slow their decline. We used five strains of each target species, except for *Perenniporia tenuis* of which we only had two, for each experiment (Supplemental Table S1). Fungi cultures of the seven species were gathered from collections (majority of Finnish strains had been in +4 °C for over 1 year in 5-ml cryo tubes, but several were taken from −80 °C storage). One Swedish strain of *Antrodia infirma* and one of *Amylocystis lapponica* had been stored in wood at +4 °C. The other Swedish strains were collected in autumn of 2022 from Swedish forests and cultured on malt yeast agar Petri plates at 20° for 1–3 weeks depending on the speed, to obtain pure cultures for use.

**Table 1:** The species used in this study. Information is provided on their most recent red-list status in Finland and Sweden and their primary natural host wood type

Species	Red-list status Fin/Swe	Regional red-list ID Fin/Swe	Species-specific wood
*Antrodia crassa*	EN/CR	MX.205424/6008693	Pine (*Pinus sylvestris*)
*Antrodia infirma*	VU/EN	MX.205426/71	Pine (*Pinus sylvestris*)
*Amylocystis lapponica*	NT/VU	MX.205412/48	Spruce (*Picea abies*)
*Skeletocutis stellae*	VU/VU	MX.206181/1505	Spruce (*Picea abies*)†
*Perenniporia tenuis*	CR/EN	MX.206147/2042	Aspen (*Populus tremula*)‡
*Radulodon erikssonii*	VU/VU	MX.205825/1347	Aspen (*Populus tremula*)
*Haploporus odorus*	VU/VU	MX.205959/760	Willow (*Salix caprea*)§

Red-list status: *CR* critically endangered, *EN* endangered, *VU* vulnerable, *NT* near threatened. †Species also less frequently found on *Pinus sylvestris*

‡Species found on other hardwoods like *Salix caprea*, *Alnus* spp., and *Betula* spp. §Species that colonize living trees. The Finland red-list status: Hyvärinen et al. (2019); Sweden red-list status: SLU Artdatabanken (2025)

Strains were newly collected from wild fruit bodies, and pure cultures were produced by placing a small piece of internal tissue onto malt-yeast agar (MYA). Clean hyphal tips were then transferred to fresh MYA plates under sterile conditions. Cultures were DNA tested prior to use in this study. For Sanger sequencing, we used PrepMan Ultra (Life Technologies Corp., Calrsbad, CA, USA) to prepare the samples, checked sequences in Geneious Prime (version 2023.0.1) and blasted against databases of UNITE and NCBI to ensure the correct species identification. The detailed procedure is described in an earlier study (Crosier et al. 2025). The strain sequences are stored in GenBank (accession numbers in Supplemental Table S1). All the living strains are maintained and stored at the Natural Resources Institute Finland (Luke) culture collection in Helsinki, Finland. Some strains are additionally deposited in the HAMBI culture collection in Helsinki, Finland (see Supplemental Table S1).

### Supplemented agar

We tested the effect of birch sawdust supplementation in agar on mycelial growth rate and ergosterol content. All strains were put on MYA to grow enough mycelium to make transfers for the first test. After enough mycelium was present in starting dishes, small myceliated agar plugs were taken using glass pipettes with a 1.0 mm diameter (product number M4230NO250TH4; Tamro Med-Lab, Vaanta, Finland) and transferred to three replicates each of two agar recipes. The first recipe used relatively standard ingredients in mushroom cultivation in the following proportions per 1 liter: 7 g malt extract (Bacto, Gibco, Life Technologies Corp., Calrsbad, CA, USA), 0.5 g yeast extract, 1 g bacteriological peptone (VWR Chemicals, Radnor, PA, USA), and 16 g bacteriological agar (Product no.: J637, VWR Chemicals, Radnor, PA, USA). The second recipe used the previous recipe as a base mixture and was further supplemented with finely powdered birch sawdust (RUF; Northumberland, UK) at 7 g per liter, which was distributed with a magnetic stirrer inside the cuvette of the MediaPrep machine (Systec GmbH; Karlsruhe, Germany).

Mycelial growth was monitored for 3 weeks in the fastest growing species (*Radulodon erikssonii* and *P. tenuis*) and 4 weeks for the slowest growing species (*Antrodia crassa*,* A. infirma*,* A. lapponica*,* Skeletocutis stellae*,* Haploporus odorus*). Every 2 or 3 days, the radius of the mycelium was measured (cm) in two directions and averaged. When the monitoring period ended, either when the mycelium reached the edge of the petri plates in fast growing species or after 4 weeks for the slower species, the mycelial dishes were placed in the −80 °C freezer. Dishes were stored in the freezer overnight and then freeze-dried for 24 h at a temperature of −88 °C and a pressure of 1.0 mbar (Alpha 2-4 LSCbasic, Martin Christ GmbH, Osterode, Germany). After freeze-drying, agar and mycelium was transferred to a sterile mortar and pestle and ground into a fine powder adding liquid nitrogen as needed. The powdered material was stored in −80 °C until investigating fungal biomass in ergosterol tests (described later).

### Temperature

We investigated the effect of temperature on mycelial growth rate. To do this, we grew every strain under a range of temperatures: 6, 12, 18, 20, 24, 28, 32, and 36.5 °C and measured growth. First, we made fresh cultures of each strain on MYA Petri dishes. There was one dish per strain per temperature. Dishes were left to grow for 1 month at the assigned temperature in the dark (or until reaching the edge of the plate), and radial growth was measured every 2–3 days in two directions and averaged. Dishes were stacked (five plates on top of each other) and were put back in a new order and place in the chamber after each measurement. Because of limited growing chambers, these tests were not all carried out simultaneously, and so new Petri plates of one strain per species were made during each round of testing and placed in 20 °C as a reference dish, to detect if worse growth could be due to temporal strain deterioration. Only one strain (*A. lapponica* JPC38) showed obvious deterioration and was dropped from the analysis, and for all others, we modelled the effect of start day (described in the “Statistical analysis” section).

### Vermiculite supplementation and gas exchange on grain spawn

We tested how vermiculite supplementation and gas exchange on grain spawn affected the growth of threatened fungi. All conditions used rye grain as a base, which was chosen for its wide-scale availability, relatively low cost, and ease of use compared to other grains, making methods widely applicable and replicable.

Grain spawn was prepared in two recipes: one with plain rye grain and the other supplemented with vermiculite (heated and expanded mica mineral with medium granulate size, Tyroler Glückspilze, Innsbruck, Austria). Vermiculite served primarily as a moisture-maintaining additive due to its ability to hold many times its own weight in water. Initially, the grain was submerged in hot water (~50 °C) and soaked overnight (approximately 18 h) to attain the desired texture and stimulate bacterial activation, thereby enhancing sterilization success. Subsequently, it is drained thoroughly to remove any excess water. For the vermiculite supplementation condition, vermiculite was moistened with hot water to “field capacity” (where a hand-squeeze test yields approximately one to two drops of water, indicating near-saturation without excessive free water) and then mixed with soaked grain at a 1:2 ratio of volume. For testing the impact of gas exchange on mycelial growth speed, in combination with vermiculite addition or not, we used two different types of Microbox grow containers manufactured from autoclave-safe polypropylene and having different microporus filter strips allowing gas exchange without contamination (Model: O95/40+OD95; SacO2, Deinze, Belgium). One of the filters had low gas exchange (white #10) and one high gas exchange (green #40). The two filters allow 9.87 and 81.35 gas exchanges per day, respectively, when measured empty. The actual values vary with the respiration rate of the fungi growing inside but are regulated by these values. Grain with or without vermiculite was loaded into Microbox grow containers (120 g per container) and then sterilized at 121 °C and 15 PSI for 1 h and 40 min. After substrates cooled, they were inoculated with a single piece of myceliated agar. Three replicates per fungal strain per growth substrate (grain with or without vermiculite) and gas exchange were made. Grow containers were arranged so that air flow was unimpeded. We measured the growth rate of the mycelium (cm per day) by taking the average of radial measurements in two directions from each agar piece every 2–3 days.

### Wood type

We tested the ability of each fungal species to grow on the preferred natural host wood (Table 1) as well as on widely available birch wood (*Betula pendula*). This test was carried out on both sawdust and wooden dowels.

Sawdust, sieved to 3.5 mm, was moistened with hot water to approximately 65% moisture and then filled into mushroom grow bags (model 4 T, Unicorn Bags, Plano, TX, USA), with each bag contained 750 g of sawdust. Six replicates were made for each strain, of which three were birch sawdust and three were the species-specific wood type (Table 1). The bags of substrate were sterilized at 121 °C and 15 PSI for 2 h and 20 min. Sawdust was inoculated with fully colonized and fresh grain spawn which had finished growing less than 3 days before use, at a rate of 5% mass, and allowed to grow at 20 *°*C until fully colonized (10–14 days depending on species and shaken once in the middle of growing to redistribute mycelium). All replicates of both wood types were stopped at the same time per strain, when mycelium was present throughout the entire substrate.

We prepared the dowels by first adding to hot water and soaking for 48 h. Then, they were drained, added to grow bags (Unicorn Bags, 10 T, Plano, TX, USA) in batches of 1000, and sterilized at 121 °C and 15 PSI for 2 h. We then placed one sterile dowel in the center of a MYA Petri plate and inoculated fungi in the same plate. Mycelial transfers were obtained by using a sterilized hollow glass rod to take 6 mm plugs from starting dishes and placing two in the plate with the dowel, one on each end, 6 mm away from the dowel. Each strain was added to three replicate Petri plates of each species-specific wood dowel and birch wood dowel (Table 1). Plates were grown at 20 °C, until the dowels were visibly completely covered in mycelium. All replicates of both wood types were stopped at the same time per strain.

When fully colonized, dowels and 10 g sawdust subsamples were moved to −80 *°*C freezer. After freezing, wood materials were freeze dried at −88 °C and a pressure of 1.0 mbar (Alpha 2-4 LSCbasic, Martin Christ GmbH, Osterode, Germany). Freeze-drying time was 72 h for dowels and 48 h for sawdust, after which samples were pulverized for ergosterol measurements (see below). Sawdust was pulverized in a TissueLyser II (QIAGEN, Hilden, Germany) at a frequency of 24 Hz for intervals of 2 min until fine powder was achieved. We first cut dowels into 6 smaller pieces and then pulverized in an IKA A10 Grinder cutting mill (IKA Labortechnik, Staufen, Germany) in intervals of 20 s, until a fine powder consistency was achieved. The mill was cooled to −10 *°*C during grinding to prevent the ergosterol from degrading. Milled wood was stored in the −80 *°*C freezer until tested for ergosterol.

### Ergosterol measurement

Ergosterol measurements were used to determine fungal biomass (Ekblad et al. 1998; Wallander et al. 2013; Adamczyk et al. 2023) in Petri plates (sawdust vs. plain) and in sawdust and dowels (different wood types). After samples were freeze dried and pulverized, they were sent for liquid chromatography-mass spectrometry, following the extraction and biomass estimation methodology described by Adamczyk et al. (2023). The results are shown as mg ergosterol per gram of dry mass (dry weight).

### Statistical analyses

All analyses were done separately for each fungal species. Analyses were carried out with the statistical program R version 4.2.1. (R Core Team 2024). For the following models, we checked the assumptions of the models (normality, potential outliers) and plotted the residuals versus the fitted values for each GAM model using *gam.check* and *plot.gam* from *mgcv* package (Wood 2017) and for each LME using the basic plot function, to verify whether model assumptions were fulfilled.

### Supplemented agar analysis

To investigate the effect of added birch sawdust on the growth of threatened fungal species on agar, we estimated two separate linear mixed effect models (LMEs) from the *nlme* package (R Core Team, Pinheiro and Bates 2024) for each species. In the first model, we used ergosterol as the variable response, and in the second one, we used linear growth (mm per day during the linear growing phase). The explanatory variable in both models was the agar media (a factor with two levels: no sawdust added to agar media or with sawdust added), and strain was included as a random factor in the models to account for observations of the same fungal strain being more similar than randomly collected fungal strains. Prior to modeling, the ergosterol content data for all species, excluding *P. tenuis* and *R. erikssonii*, were log-transformed to achieve better model fit. Predicted values and standard errors for linear growth and ergosterol (back transformed if applicable) were calculated for using the predictSE.lme function from the *nlme* package (R Core Team, Pinheiro and Bates 2024). We used the predicted values to draw results plots with *ggplot2* (Wickham 2016).

### Temperature analysis

To measure the effect of temperature on linear growth rate (average cm of radial growth per day during the linear growing phase), we used linear mixed-effects models (LMEs) where linear growth was the response variable and the explanatory variables were (1) temperature (12, 18, 20, 28, 32, and 36.5) and (2) day of the year which the test was started (assuming that this might affect the growth rate), with strain as a random effect (except for *R. erikssonii*, for which strain had no effect). The base temperature to make comparisons was set at 24 °C. Temperatures where no strains of a given species grew at all, or only had one or two observations of growth for the specific species at the end of the observation period, were not included in the models. The linear mixed-effects models were estimated using the *nlme* package (R Core Team, Pinheiro and Bates 2024). The linear growth data for *P. tenuis* were log transformed to achieve normality. Predictions from the linear mixed-effects model were generated using the *predict* function within the *nlme* package, and these predictions were used to create the plots describing the growth rate of each threatened fungal species as a function of temperature.

To demonstrate more detail of the effect of temperature on mycelial growth, we modelled each temperature individually, using generalized additive mixed models (GAMMs) with the *gamm4* package (Wood and Scheipl 2020), where growth (average radius in mm) was our response variable, and the explanatory variables were (1) temperature (12, 18, 20, 24, 28, 32, and 36.5) and (2) the time of measurement (days from the start of the experiment as a smoothed variable), with a random effect of strain. Growth in this case was total growth, not only the linear phase, so predicted plots show growth from the beginning to end, encompassing lag time before growth started and when it started to taper off. Predictions for each individual temperature model were generated using the *predict.gam* function from the *mgcv* package (Wood 2017) on the GAM component of the models, based on new data frames created with *seq* and *rep* functions to ensure smooth curves. These predictions represented growth over time and were plotted using the *ggplot2* package (Wickham 2016).

### Vermiculite supplementation and gas exchange on grain spawn analysis

For determining effects of vermiculite addition and gas exchange rate on mycelial growth rate (mm per day), we used linear mixed-effects (LMEs) models using the *nlme* package (R Core Team, Pinheiro and Bates 2024) with an interaction effect between two explanatory variables: (1) media (a factor with two levels: plain rye grain and rye grain with vermiculite) and (2) gas filter (a factor with two levels: low gas exchange and high gas exchange). Fungal strain was included as a random factor in the models. For some fungal species (*S. stellae*, *A. lapponica*, and *H. odorus*), log transformation was used to achieve normality of the responses (mycelial growth rate). Predictions from the linear mixed-effects models were generated using the *predictSE.lme* function from the *AICcmodavg* package (Mazerolle 2023), which provided predicted values and standard errors. These standard errors were used to calculate upper and lower limits for the predicted values. The *exp* function in base R (R Core Team 2024) was used to back-transform the log-transformed predictions to the original scale. Plots were created using base R graphics functions.

### Wood type analysis

To investigate the effects of wood quality on mycelial growth on sawdust spawn and dowels, we estimated linear mixed-effects models (LMEs) using the *nlme* package (Pinheiro et al. 2023, R Core Team 2024). Ergosterol content was used as the response variable, wood type (a factor with two levels: birch or typical host tree) was the explanatory variable, and fungal strain was included as a random factor. Linear mixed-effects models were fitted and result figures drawn based on the predicted values from the estimated models.

## Results

### Supplemented agar

**Fig. 1:**
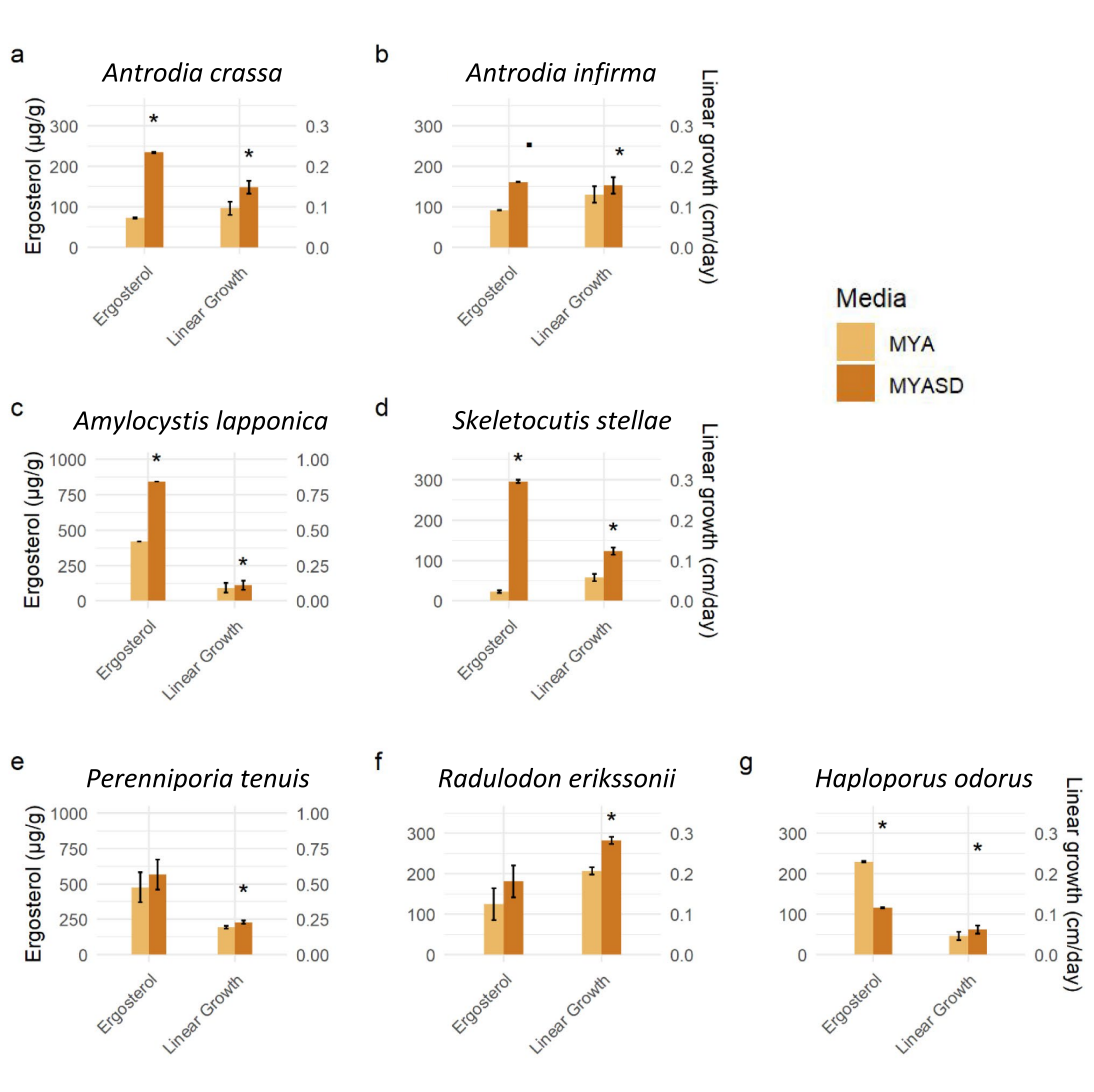
Effect of sawdust addition in agar Petri plateson mycelium growth. The figure shows predicted values of sawdust added agar compared to malt yeast agar without sawdust on the linear growth rate of mycelium (cm per day) and ergosterol content (mg ergosterol/g, dry weight) of threatened wood-inhabiting fungal species. Predicted mean values and standard errors based on the estimated models are shown. Significant results are marked with “*.” Indicative results are marked with “ ·” Note different axis scales. See Table 3

The addition of powdered birch sawdust had a diverse effect across fungal species, but the majority of these responses were statistically significantly positive (Table 2, Fig. 1). Linear growth rate (mm day^−1^) was significantly increased by sawdust addition in all species. The biggest positive effect on linear growth rate was in *R. erikssonii* and *S. stellae* (*p* < 0.001 for both species). Ergosterol content (fungal biomass) significantly increased in three out of the seven species. Large positive effect was seen in *A. crassa* (*p* = 0.010), *A. lapponica* (*p* < 0.001), and *S. stellae* (*p* = 0.033), but there was a significant decreasing effect in *H. odorus* (*p* = 0.006).

**Table 2:** The effect of birch sawdust supplementation in agar on growth of seven fungal species

Species	*N*	Response	Intercept		MYA + sawdust	
			Coeff. ± SE	*p*	Coeff. ± SE	*p*
***Antrodia crassa***	30	Ergosterol	4.287 ± 0.479	< 0.001	1.171 ± 0.416	0.010
		Linear growth (cm/day)	0.010 ± 0.016	< 0.001	0.053 ± 0.020	0.014
***Antrodia infirma***	30	Ergosterol	4.512 ± 0.630	< 0.001	0.571 ± 0.324	0.091
		Linear growth (cm/day)	0.130 ± 0.021	< 0.001	0.023 ± 0.011	0.043
***Amylocystis lapponica***	30	Ergosterol	6.039 ± 0.249	< 0.001	0.699 ± 0.115	< 0.001
		Linear growth (cm/day)	0.093 ± 0.033	0.009	0.017 ± 0.004	0.001
***Skeletocutis stellae***	30	Ergosterol	3.146 ± 1.293	0.023	2.543 ± 1.125	0.033
		Linear growth (cm/day)	0.058 ± 0.009	< 0.001	0.066 ± 0.012	< 0.001
*Radulodon erikssonii*	30	Ergosterol	125.25 ± 39.65	0.004	55.85 ± 46.47	0.241
		Linear growth (cm/day)	0.208 ± 0.001	< 0.001	0.075 ± 0.001	< 0.001
*Perenniporia tenuis*	10	Ergosterol	476.71 ± 105.61	0.002	88.64 ± 83.77	0.318
		Linear growth (cm/day)	0.193 ± 0.011	< 0.001	0.036 ± 0.014	0.033
***Haploporus odorus***	30	Ergosterol	5.44 ± 0.32	< 0.001	−0.68 ± 0.23	0.006
		Linear growth (cm/day)	0.046 ± 0.010	< 0.001	0.016 ± 0.005	0.002

Model coefficients (Coeff.) with standard errors of mean (SE) are presented, and MYA + sawdust shows the effect of added sawdust when compared to standard MYA (malt yeast agar without sawdust). *N* is the number of observations for each response per species. Statistically significant results (Coeff. ± SE, *p* < 0.05) are in bold, and indicative (0.05 ≤ *p* < 0.10) have been underlined. Species are in bold if the data was log transformed in the ergosterol model (see Fig. 1)

### Temperature

Temperature significantly influenced the linear growth in various species (Table 3). Compared to 24 °C, *P. tenuis* displayed significant positive effects at 28 °C (*p* = 0.001) and 32 °C (*p* < 0.001) and negative effects at 12 °C (*p* < 0.001). *R. erikssonii* also showed significant negative effects at 12 °C (*p* < 0.001), 20 °C (*p* = 0.016), and 36.5 °C (*p* < 0.001**)**. *A. infirma* demonstrated a significant negative effect at 32° C (*p* = 0.001) and 36.5 °C (*p* = 0.009). Some species, *A. crassa*, *H. odorus*, *A. lapponica*, and *S. stellae* were more temperature limited so that higher temperatures were not able to be modelled due to lack of growth. Of these, *S. stellae* exhibited the least significant overall results within the range of 12–28 °C. None of our species showed a significant effect of start day.

**Table 3:** Temperature effect on mycelial growth rate

Species	N	Intercept		Start day		12 °C		18 °C		20 °C		28 °C		32 °C		36.5 °C	
		Coeff. ± SE	*p*	Coeff. ± SE	*p*	Coeff. ± SE	*p*	Coeff. ± SE	*p*	Coeff. ± SE	*p*	Coeff ± SE	*p*	Coeff. ± SE	*p*	Coeff. ± SE	*p*
*Antrodia crassa*	21	0.14 ± 0.03	< 0.001	−1.5e−4 ± 1.9e−4	0.461	-	-	−0.04 ± 0.03	0.241	−0.08 ± 0.03	0.039	−0.01 ± 0.03	0.764	−0.02 ± 0.03	0.495	-	-
*Antrodia infirma*	37	0.17 ± 0.02	< 0.001	−1.6e−4 ± 1.0e−4	0.157	−0.06 ± 0.04	0.134	−0.04 ± 0.06	0.137	0.14 ± 0.03	0.889	−0.02 ± 0.03	0.414	−0.11 ± 0.03	0.001	−0.11 ± 0.04	0.009
*Amylocystis lapponica*	21	0.05 ± 0.01	0.001	−2.2e−4 ± 1.3e−4	0.127	0.040 ± 0.03	0.197	0.02 ± 0.02	0.326	0.06 ± 0.02	0.041	0.02 ± 0.02	0.382	-	-	-	-
*Skeletocutis stellae*	24	0.02 ± 0.02	0.265	−3.6e−4 ± 3.6e−4	0.343	0.07 ± 0.07	0.348	0.05 ± 0.03	0.120	0.09 ± 0.06	0.173	0.03 ± 0.03	0.420	-	-	-	-
***Perenniporia tenuis***	16	−1.45 ± 0.07	< 0.001	5.0e−4 ± 5.2e−4	0.370	−1.20 ± 0.14	< 0.001	0.11 ± 0.10	0.279	−0.18 ± 0.09	0.113	0.34 ± 0.10	0.001	0.68 ± 0.10	< 0.001	−0.17 ± 0.14	0.370
*Radulodon erikssonii*	35	0.71 ± 0.06	< 0.001	−5.7e−5 ± 8.5e−4	0.947	−0.47 ± 0.08	< 0.001	−0.25 ± 0.08	0.132	−0.24 ± 0.08	0.016	0.08 ± 0.15	0.629	0.18 ± 0.16	0.296	−0.52 ± 0.08	< 0.001
*Haploporus odorus*	26	0.04 ± 0.01	0.003	2.2e−4 ± 1.3 e−4	0.123	−0.09 ± 0.01	0.012	−0.03 ± 0.02	0.121	−0.01 ± 0.02	0.623	−0.06 ± 0.02	0.01	-	-	-	-

Results from LME models showing the effect of time (start day within a year) and temperature (compared to 24 °C) on the linear growth rate (cm/day) of threatened wood-inhabiting fungal species. Values represent model coefficients (Coeff.) with standard errors of mean (SE). *p*-values are provided. N represents the number of observations for each species. Dashes (-) indicate that the effect was not modelled due to lack of growth. The growth data for species in bold were log transformed before modelling

The GAM temperature models (Supplemental Fig. S1) show more detail about the entire growth period and give a more holistic picture. For example, while some of the linear growth phases were not significantly different in the LME models, the start of the linear growth phase clearly started sometimes earlier or later.

### Vermiculite supplementation and gas exchange on grain spawn

**Fig. 2:**
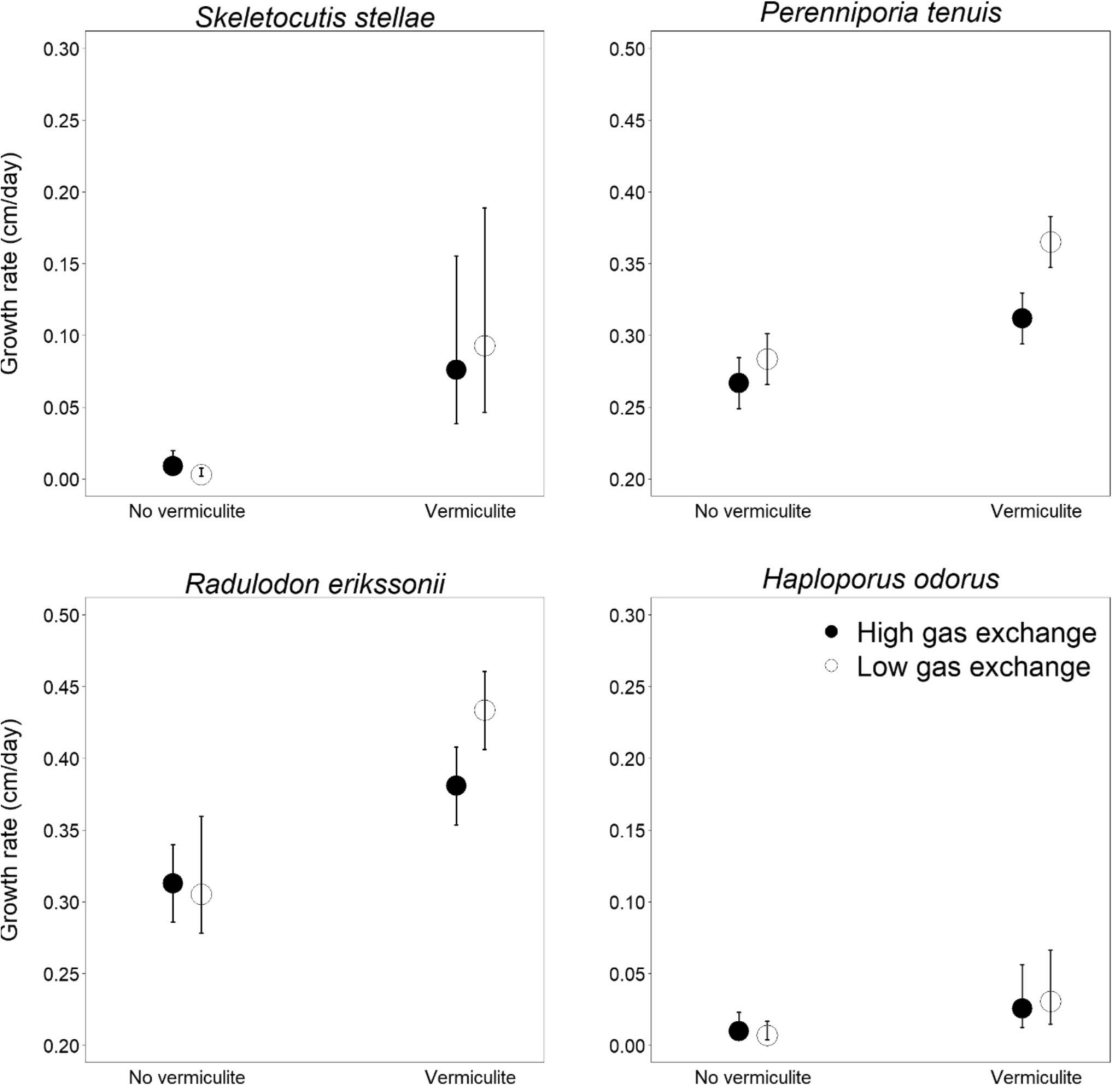
The effect of vermiculite addition and gas exchange (high or low) on the growth rate of mycelium. All species shown in the figure demonstrated a significant positive effect on the growth rate (cm per day) from vermiculite addition when grown on rye grain. Gas exchange rates did not have a statistically significant impact. Predicted mean values and standard errors based on the estimated modes are shown. See Table 4

Four of the species (*S. stellae*,* P. tenuis*, *R. erikssonii*, and *H. odorus*) demonstrated a significant positive response in growth rate to the addition of vermiculite to the grain substrate (Fig. 2, Table 4). No species had a significant impact from gas exchange rate, and no interaction effect was found between gas exchange and substrate composition.

**Table 4:** Effects of vermiculite and gas exchange

Species	*N*	Intercept		Media: with vermiculite		Gas exchange: low		Interaction: Vermiculite + low air	
		Coeff. ± SE	*p*	Coeff. ± SE	*p*	Coeff. ± SE	*p*	Coeff. ± SE	p
*Antrodia crassa*	30	0.040 ± 0.008	< 0.001	0.003 ± 0.006	0.646	0.0113 ± 0.006	0.055	0.001 ± 0.008	0.935
*Antrodia infirma*	30	0.263 ± 0.033	< 0.001	0.011 ± 0.021	0.616	−0.017 ± 0.021	0.415	0.035 ± 0.030	0.242
***Amylocystis lapponica***	30	−3.861 ± 0.971	< 0.001	0.395 ± 0.433	0.365	−0.625 ± 0.433	0.155	0.733 ± 0.612	0.237
***Skeletocutis stellae***	30	−4.876 ± 0.701	< 0.001	2.065 ± 0.519	< 0.001	−0.974 ± 0.519	0.066	1.169 ± 0.734	0.117
*Radulodon erikssonii*	30	0.313 ± 0.027	< 0.001	0.068 ± 0.034	0.049	−0.007 ± 0.034	0.828	0.060 ± 0.048	0.213
*Perenniporia tenuis*	10	0.267 ± 0.018	< 0.001	0.045 ± 0.019	0.030	0.017 ± 0.019	0.394	0.037 ± 0.027	0.191
***Hapoloporus odorus***	30	−4.815 ± 0.752	< 0.001	0.899 ± 0.362	0.016	−0.321 ± 0.362	0.379	0.491 ± 0.511	0.341

Results of linear mixed-effects models showing the effects of vermiculite addition (media: with vermiculite), gas exchange rate (gas exchange: low), and their interaction on mycelial growth rate for threatened wood-inhabiting fungal species. Statistically significant results (*p* < 0.05), i.e., coefficient estimates (Coeff.) ± standard errors of means (SE), and *p*-values are in bold, and indicative (0.05 ≤ *p* < 0.10) are underlined. *N* indicates sample size. Species in bold were transformed to log scale for analysis

### Wood type

The effect of wood type on ergosterol content varied between sawdust and dowels (Table 5). In sawdust, wood type only influenced *H. odorus*, which had significantly (*p* ≤ 0.05) higher ergosterol content on the host wood (willow) than on birch. Three species were affected by wood type on dowels: *A. crassa* and *S. stellae* had significantly higher ergosterol content on birch dowels, whereas *A. lapponica* had a higher content on the host wood (spruce) dowels.

**Table 5:** Effects of wood type on ergosterol

Species	*N*	Media	Intercept		Host wood	
			Coeff. ± SE	*p*	Coeff. ± SE	*p*
*Antrodia crassa*	30	Sawdust	79.60 ± 10.69	< 0.001	17.37 ± 9.38	0.076
		Dowels	74.64 ± 10.57	< 0.001	−32.46 ± 14.94	0.040
*Antrodia infirma*	30	Sawdust	151.29 ± 62.36	0.025	46.81 ± 36.39	0.214
		Dowels	29.43 ± 4.20	< 0.001	−10.32 ± 5.91	0.094
*Amylocystis lapponica*	30	Sawdust	172.69 ± 61.28	0.010	−32.90 ± 26.44	0.226
		Dowels	47.67 ± 20.94	0.048	27.92 ± 10.81	0.017
*Skeletocutis stellae*	30	Sawdust	203.89 ± 119.32	0.113	−59.32 ± 41.41	0.178
		Dowels	48.59 ± 21.58	0.034	−13.46 ± 6.46	0.048
*Perenniporia tenuis*	10	Sawdust	64.46 ± 45.74	0.192	110.66 ± 63.00	0.112
*Radulodon erikssonii*	30	Sawdust	178.00 ± 45.48	0.001	32.57 ± 16.74	0.064
		Dowels	200.88 ± 50.84	0.001	−0.63 ± 29.78	0.984
*Hapoloporus odorus*	30	Sawdust	248.90 ± 15.47	< 0.001	62.36 ± 15.35	< 0.001
		Dowels	329.31 ± 38.50	< 0.001	−18.75 ± 19.29	0.347

The effect of wood type (in both sawdust and dowel form) on ergosterol content of threatened wood-inhabiting fungi (fungal biomass, mg per g dry weight). The effect of natural host wood compared to birch is presented. Statistically significant (*p* < 0.05) coefficients (Coeff.) and standard errors of mean (SE) are in bold, and indicative (0.05 ≤ *p* < 0.10) have been underlined

## Discussion

**Fig. 3:**
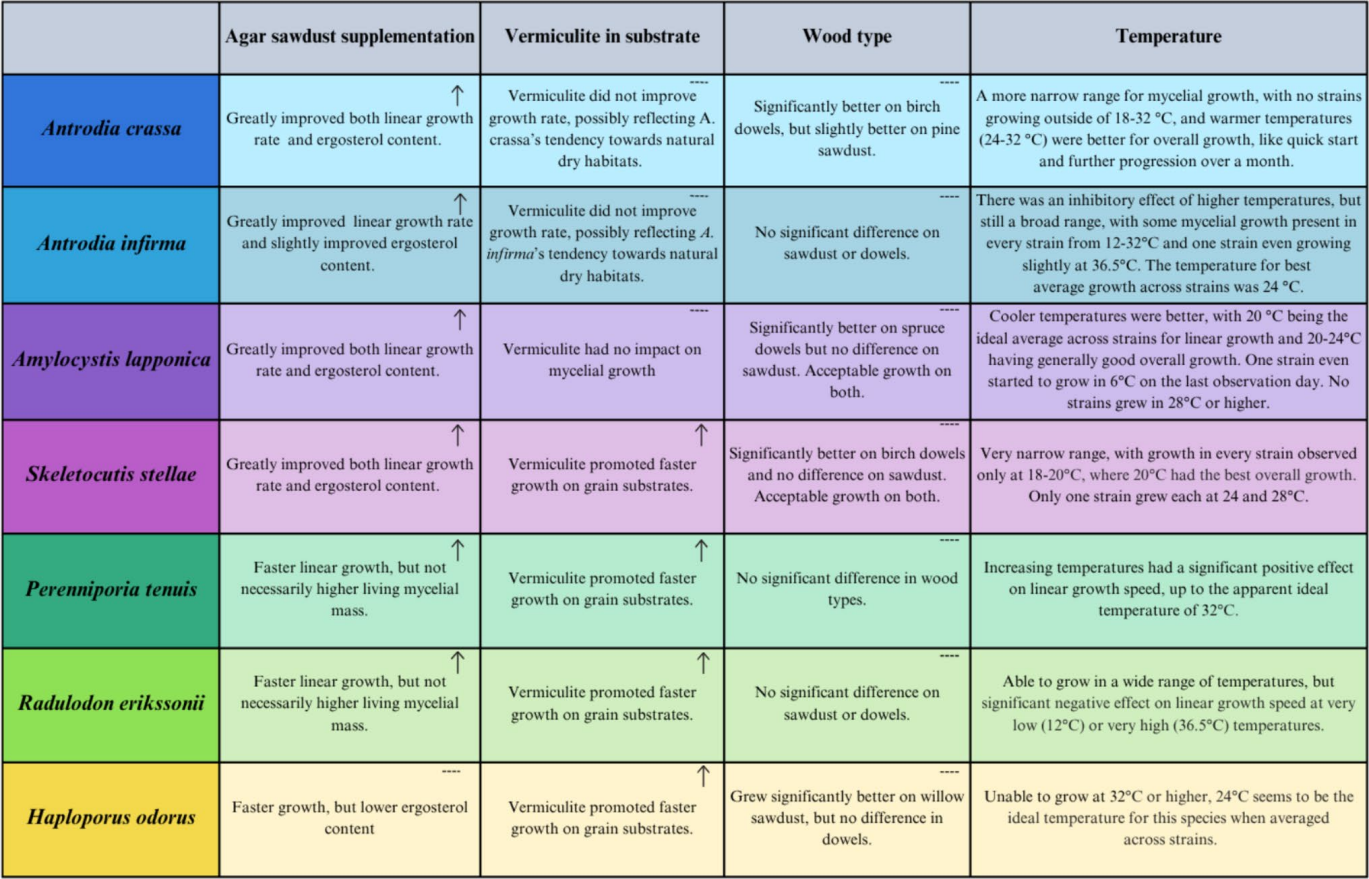
A summary of the effects of each experiment on the threatened fungi used in this study. A “**↑** indicates a positive effect from the treatment, where a “---” indicates neutral or mixed results. None of our treatments had a negative effect for any species

Our experiments aimed to explore techniques that could improve the growth success of certain threatened wood-inhabiting fungi, particularly in the early stages of cultivation. As expected, the interventions did not impact all species equally, but we are able to provide valuable insights for cultivating challenging fungal species, either for conservation purposes or other applications requiring bulk mycelium spawn production. A summary of the impacts of each intervention by species is provided in Fig. 3.

Our first hypothesis was that adding wood sawdust to agar would enhance mycelial growth. In most cases, this hypothesis was confirmed, with wood supplementation leading to improved growth. Wood is a more complex nutrient source than most simple agar recipes, and fungi express a spectrum of enzymes to break down wood components such as lignin and cellulose (Andlar et al. 2018). We further hypothesized that the slower-growing and more challenging species would show a greater difference between conditions, and this was also largely supported by our results. For example, the relatively less challenging to grow aspen-associated species, *P. tenuis* and *R. erikssonii*, did not show significantly improved ergosterol content when wood was added, whereas *S. stellae*, the most challenging to grow species, benefitted greatly (overall an average of 45% increased ergosterol when sawdust was added).

In our second hypothesis, we expected that temperature would have a highly variable impact by species. This was verified, and each species’ optimal temperature and viable range are detailed below by species. In cases where natural microclimate preferences are known, optimal temperatures in the lab seemed to reflect these natural preferences. *P. tenuis*, known to fruit in open sunny areas (Martikainen et al. 2000), grew better at higher lab temperatures, whereas *A. lapponica*, found almost exclusively in old spruce forests (Kunttu 2007; Runnel et al. 2020) which are associated with cooler temperatures, grew better at a lower temperature. On the other hand, *A. crassa* also grows in open sunlit conditions, indicating that the effect of temperature is complex, probably interacting with other factors in structuring fungal communities under natural conditions (Edman et al. 2021). Such natural temperature-related trends are not known for many of the species used here, so stronger conclusions cannot be drawn, but it is interesting to note for future studies considering how to best grow rare fungi. Also, while we found no impact on growth from what day of the year tests were started.

We also hypothesized that the addition of vermiculite would speed up mycelial growth. This was confirmed for four of the seven species tested, and interestingly, the effect seemed to be linked to natural microclimate preference. Species that grew faster with vermiculite addition include *P. tenuis*, found mostly on trees laying on moist ground (Martikainen et al. 2000); *R. erikssonii*, found in moist forests during our strain collection; and *H. odorus*, which, while growing in the drier microclimate of heartwood, is also associated with wet and humid habitats (Kotiranta and Niemelä 1996). Species adapted to drier environments, like those growing on kelo logs (*Antrodia* species) showed no significant improvement. We expected an interaction effect between higher gas exchange, which can lead to quicker substrate drying, and moisture-holding vermiculite to possibly counteract that; however, no interaction was observed. We can generally recommend adding vermiculite when attempting to produce larger amounts of spawn for challenging fungal species, as effects ranged from neutral to positive with no negative effects that we found. Additionally, it extends the moisture stability of the substrate when producing bulk over time and prevents slow-growing species from drying out before colonization.

Finally, we hypothesized that alternative wood substrate could yield comparable or better results than the fungi’s natural host wood, particularly for the purpose of bulk mycelium spawn production. This was generally confirmed, as in most species, no difference was found between wood types, and in some species, they grew better on dowels made from birch. Possible explanations for this could be that birch wood’s higher levels of easily accessible nutrients, relatively more porous structure, and lower levels of certain antimicrobial extractives compared to pine and spruce (Routa et al 2017) facilitate faster colonization by most wood-inhabiting fungi in the absence of competition. The idea is supported by a recent study that recommended birch (*Betula*) wood for dowel spawn because it had the fastest growth rate of *Inonotus obliquus* than different wood substrates, regardless of a strain’s original host tree species (Adamson et al 2023). We suggest that for large-scale fungal cultivation, non-host wood substrates may be more cost-effective while still supporting effective growth, as is the common practice in edible mushroom production.

Our findings have practical implications for both conservation and biotechnology. The growth observed on alternative substrates demonstrates a method for reducing production costs of fungal spawn for conservation efforts. Beyond conservation, our results also address key limitations in emerging wood-based biotechnologies. A common criticism of fungal composites is their slow manufacturing process (Jones et al. 2020). Similarly, a constraint for biopulping is the slow reaction rate and the risk of contamination during the colonization phase (Singh et al. 2010). Our methods, which focus on reducing spawn run time, provide some approaches to accelerate the production cycle. The positive effect of vermiculite highlights a simple, cost-effective way to improve growth and prevent substrate desiccation, a common challenge in large-scale solid-state fermentation. By reducing the time required for mycelial colonization, our methods can contribute to these emerging technologies.

## Considerations and limitations

In addition to the main findings, there are several important considerations and limitations that must be addressed in future research. We measured both linear growth and ergosterol content as indicators of mycelial vigor, as it is important to note that fast growth alone is not always healthy. In some species, increased extension rate occurs with less branching density (Jones et al. 2017). Additionally, ergosterol has limitations as a stand-in for fungal biomass. Despite its frequent use in estimating fungal quantity (Gessner 2020), researchers have long cautioned against ergosterol’s limitations as a direct proxy for fungal biomass because of its variability across species, mycelial age, and culture conditions (Bermingham et al. 1995; Charcosset and Chauvet 2001). Our study only made intraspecies comparisons using consistent-aged cultures, thereby limiting ergosterol content variation. Although varied culture media could influence the ergosterol-to-biomass ratio, visual assessment strongly indicated that higher ergosterol readings corresponded to greater fungal biomass in Petri dishes and sufficient biomass on wood substrates for our study’s objectives. For future studies, it may be useful to incorporate additional physiological markers such as chitin, used as a structural indicator, in combination with metabolic markers such as ATP or respiration, to better understand the full picture of mycelial health (Nilsson and Bjurman 1998; Abelho 2005; Eikenes et al. 2005).

Our study used sawdust as an addition to agar, but different quantities or additives were not explored. We used only birch sawdust, but other wood types can have a less desirable effect for some fungi (Cortina-Escribano et al. 2020), and other additives might also enhance growth, especially if food sources are changed regularly to prevent strain senescence. It is valuable to investigate the effects of other agar supplements.

While we hypothesized that vermiculite could influence growth by retaining moisture, we did not explicitly control for moisture content in the substrates, as the scope here was practical application. Future studies could investigate moisture levels more precisely, as minimum moisture thresholds vary by species (Brischke and Alfredsen 2020). As moisture decreases during substrate colonization (Bandura et al. 2022), substrates could be prepared to various specific moisture contents and measured after growth to quantify the moisture lost in the vermiculite versus the non-vermiculite substrates. Similarly, we did not measure gas exchange. Filters only controlled the maximum possible exchange, and a more controlled environment could help clarify the role of gas exchange in fungal growth. Future experiments could benefit from directly controlling oxygen and CO₂ levels, testing a range of mixes to determine optimal conditions for different species by using a vacuum to vent chambers based on sensor readings (Chung 2021).

Finally, it is important to note that we did not analyze enzyme production or other metabolic markers, which provide a fuller understanding of mycelial response to wood (Boyle and Kropp 1992). Instead, we focused on growth rate and ergosterol as indicators of mycelial mass for bulk spawn production. Metabolic activities and genomic expression can be influenced by wood substrate type (Daly et al. 2018; Pawlik et al. 2019), and these responses may be important depending on the application, warranting further investigation for uses beyond fungal species introduction.

## Conclusion

All fungal species, and even strains, have their own ideal growing conditions, but we present certain strategies for each stage of the cultivation process which are worth trying when attempting to cultivate challenging fungal species. For some species it is difficult to get enough mycelium even at the Petri dish phase, perhaps especially after reculturing from long-term storage or due to partial strain senescence. The addition of certain ingredients such as those described in this paper, with the aim of improving nutritional complexity or moisture content, can improve mycelial growth making it possible to produce enough inoculum for fungal applications. Wood type choice might not be the most critical consideration when producing spawn for certain fungal applications (e.g., reintroduction), in case there are more cost-effective options when designing a project. There seems to be indication (from temperature and moisture retention in substrates) that known microclimate preferences could provide a reasonable starting point for lab conditions. When working with fungal species new to cultivation, one should become familiar with the mycelial characteristics under various growth conditions. These methods may also prove valuable in both long-term fungal collection maintenance and biotechnological settings, for example, in the production of desirable metabolites. Such metabolites have been examined in some of the fungal genera within this study (Sum et al. 2023), and *H. odorus* itself has a long history of ethnomycological importance (Zmitrovich et al. 2019), highlighting the broader applications that could benefit from improved cultivation techniques of rare or challenging fungi.

## Supplementary Information

Below is the link to the electronic supplementary material.

ESM (PDF 548 KB)

## Data Availability

DNA sequences of strains used in this study can be accessed through GenBank with the accession codes PV796022 - PV796052, KC595895.1, and KY415963.1. Individual strain accession codes are listed in Supplemental Table S1.
