# Supplementary material for: Optimizing laboratory cultivation of wood-inhabiting fungi with emphasis on applied conservation
Source: Appl Microbiol Biotechnol. 2025 Sep 30;109:210. doi: 10.1007/s00253-025-13603-1 (PMC12484318; doi:10.1007/s00253-025-13603-1)

Supplemental Material

Journal: Applied Microbiology and Biotechnology

Optimizing laboratory cultivation of wood-inhabiting fungi with emphasis on applied conservation

Joette Crosier[1*], Lorin von Longo-Liebenstein[4], Mattias Edman[3], Sylwia Adamczyk[2], Leena Hamberg [1]

[1] Forest Health and Biodiversity Unit, Natural Resources Institute Finland (Luke), Latokartanonkaari 9, 00790 Helsinki, Finland

[2] Soil Ecosystems Unit, Natural Resources Institute Finland (Luke), Latokartanonkaari 9, 00790 Helsinki, Finland

[3] Department of Natural Science, Design and Sustainable Development, Mid Sweden University, Mittuniversitetet, Holmgatan, 852 30 Sundsvall, Sweden

[4] Kettula, Finland

* Corresponding author. Email: joette.crosier@luke.fi

Supplemental Table S1

Information on threatened wood-inhabiting fungal strains used in this study, including the country and locality of original specimen collection, collector, and collection date. All strains are deposited in the Biobank of Natural Resources Institute Finland (Luke) in Helsinki Finland, and additionally strains which are FBCC are deposited in Microbial Domain Biological Resource Centre HAMBI in Helsinki, Finland.

Species	Strain ID	GenBank accession	Country of origin	Collection location	Collector	Collection date
Antrodia crassa	Rescra2	PV796029	Sweden	Lill-Aha berget, Lappland	Isak Vahlström	12/10/2022
	Rescra1	PV796028	Sweden	Stor-Mullberget, Hälsingland	Mattias Edman	2/9/2022
	JPC171	PV796045	Finland	Korvua	Jorma Pennanen	4/10/2018
	OMC 1865	PV796050	Finland	unknown	Otto Miettinen	unknown
	JPC178	PV796046	Finland	Korvua	Jorma Pennanen	4/10/2018
Antrodia infirma	Neoinf4	PV796026	Sweden	Ensjölokarna, Hälsingland	Mattias Edman	2/9/2022
	Neoinf1	PV796027	Sweden	Ruokovare, Lappland	Mattias Edman	18/9/2008
	JPC17	PV796037	Finland	Hukkajärvi	Jorma Pennanen	9/3/2018
	OMC 2423	KC595895.1	Finland	unknown	Otto Miettinen	4/7/2012
	JPC54	PV796039	Finland	Hukkajärvi	Jorma Pennanen	9/4/2018
Amylocystis lapponica	Amylap1	PV796033	Sweden	Älgsmyrberget, Medelpad	Mattias Edman	24/9/2009
	Amylap3	PV796031	Sweden	Bjursberget, Medelpad	Mattias Edman	3/10/2022
	Amylap4	PV796030	Sweden	Lögdö bruk, Medelpad	Mattias Edman	3/10/2022
	JPC160	PV796044	Finland	Vepsä	Jorma Pennanen	29/9/18
	JPC38	PV796038	Finland	Vepsä	Jorma Pennanen	2/9/2018
Skeletocutis stellae	Skeste3	PV796023	Sweden	Ensjölokarna, Hälsingland	Mattias Edman	2/9/2022
	Skeste2	PV796022	Sweden	Ensjölokarna, Hälsingland	Mattias Edman	2/9/2022
	FBCC 2659	PV796036	Finland	Hyrynsalmi	Otto Miettenen / HAMBI	20/6/2019
	FBCC 2222	PV796052	Finland	unknown	HAMBI	31/12/2012
	JPC185	PV796047	Finland	Kuru	Jorma Pennanen	10/9/2018
Perenniporia tenuis	JPC67	PV796040	Finland	Säimen	Jorma Pennanen	11/9/2018
	JPC79	PV796042	Finland	Säimen	Jorma Pennanen	14/9/2018
Radulodon erikssonii	Raderi2	PV796034	Sweden	Snöberget, Norrbotten	Mattias Edman	22/9/2022
	Raderi1	PV796035	Sweden	Skovelliden, Lappland	Isak Vahlström	4/9/2022
	FBCC 144	KY415963.1	Finland	unknown	Pekka Oivanen / HAMBI	16/9/2017
	JPC70	PV796041	Finland	Säimen	Jorma Pennanen	11/9/2018
	JPC121	PV796043	Finland	Padasjoki	Jorma Pennanen	21/9/2018
Haploporus odorus	Hapodo1	PV796025	Sweden	Mycksjön,Medelpad	Mattias Edman	2022
	Hapodo2	PV796024	Sweden	Svarttjärnen, Medelpad	Mattias Edman	2022
	JPC216	PV796048	Finland	unknown	Jorma Pennanen	23/10/2018
	JPC220	PV796049	Finland	Rautalampi	Jorma Pennanen	23/10/2018
	FBCC 965	PV796051	Finland	Ylläs	Terhi Hakala / HAMBI	31/08/1999

Supplemental Fig. S1: Predicted growth over time for individual temperatures (12-36.5°C) by species, to show overall growth trend including lag time, predicted using Generalized Additive Mixed Models (GAMMs) with 'day' as a smooth term and 'Strain' as a random effect. A Tweedie distribution was used to model growth. Temperatures with no observed growth are not plotted. The figure shows predicted growth (mm) over time.

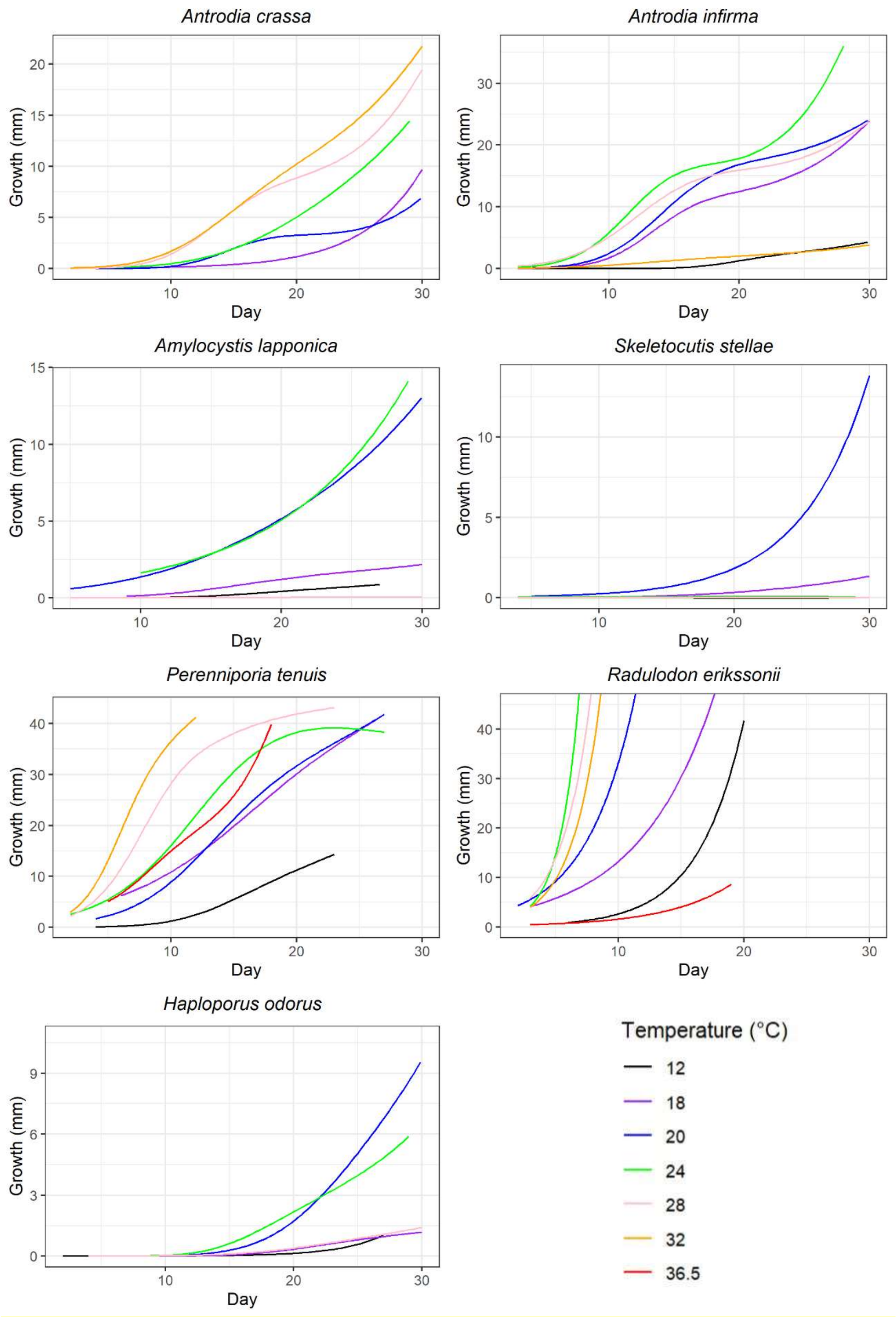

Supplement: Supplementary file 1 [file 253_2025_13603_MOESM1_ESM.pdf]
